# Health services access, utilization, and barriers for Arabic-speaking refugees resettled in Connecticut, USA

**DOI:** 10.1186/s12913-022-08733-5

**Published:** 2022-11-11

**Authors:** Ali Elreichouni, Sarah Aly, Kaitlin Maciejewski, Islam Salem, Noah Ghossein, M. Salah Mankash, James Dziura, Hani Mowafi

**Affiliations:** 1 Yale School of Medicine, Yale University, New Haven, CT USA; 2 Department of Emergency Medicine, St. Joseph’s University Medical Center, Paterson, NJ USA; 3 Yale Center for Analytic Sciences, Yale University, New Haven, CT USA; 4 Department of Emergency Medicine, Yale University, 464 Congress Ave, Suite 260, New Haven, CT 06519 USA; 5 School of Medicine, University of California-Riverside, Riverside, CA USA; 6 Yale School of Public Health, Yale University, New Haven, CT USA

**Keywords:** Refugee health, Access to health services, Patient satisfaction

## Abstract

**Background:**

Arabic-speaking refugees are the largest group of refugees arriving in the United States since 2008, yet little is known about their rates of healthcare access, utilization, and satisfaction after the end of the Refugee Medical Assistance (RMA) period.

**Methods:**

This study was a cross-sectional observational study. From January to December 2019, a household survey was conducted of newly arrived Arabic-speaking refugees in Connecticut between 2016 and 2018. Households were interviewed in Arabic either in person or over the phone by one of five researchers. Descriptive statistics were generated for information collected on demographics, prevalence of chronic conditions, patterns of health seeking behavior, insurance status and patient satisfaction using the Patient Satisfaction Questionnaire (PSQ-18).

**Results:**

Sixty-five households responded to the survey representing 295 Arabic-speaking refugees – of which 141 (48%) were children. Forty-seven households (72%) reported 142 chronic medical conditions among 295 individuals, 62 persons (21%) needed daily medication, 285 (97%) persons were insured. Median patient satisfaction was > 4.0 out of 5 for 6 of 7 domains of the PSQ-18 but wide variation (scores from 1.0 – 5.0).

**Conclusion:**

Arabic-speaking refugees in Connecticut participating in this study were young. The majority remained insured after their Refugee Medical Assistance lapsed. They expressed median high satisfaction with health services but with wide variation. Inaccessibility of health services in Arabic and difficulty obtaining medications remain areas in need of improvement.

**Supplementary Information:**

The online version contains supplementary material available at 10.1186/s12913-022-08733-5.

## Background

The United Nations High Commission for Refugees (UNHCR) defines refugees as people who have crossed an international border in search of safety from war, violence, conflict, or persecution [1].The United States has received approximately three million refugees since 1975, the demographics of which vary with global events and trends [2]. As a result of recent conflicts in the Middle East and North Africa (MENA), Arabic-speaking refugees comprised the largest group arriving in the United States between 2008 and 2020 [2].

### Challenges of researching Arabic-speaking refugees as a group

There is insufficient research on the physical and mental health needs of Arab-Americans overall, despite large communities in the United States for well over a century [3–5]. The study of this ethnic community’s health in the United States is complicated by the lack of an ethnicity identifier in standardized public surveys, vital statistics, and most electronic medical records. “Arab” or “Middle Eastern” ethnicity is subsumed under “White/Caucasian” which inhibits the use of large datasets to detect disparities in outcomes and access that affect community health [6]. Furthermore, recent events including global wars, terrorist attacks, and the political climate in the United States has led to a rise in Islamophobia and stigmatization of not only Muslims but of people suspected, either through language or appearance, to be from Muslim countries [3, 7–9]. While the Arabic language is spoken in a wide range of countries that exhibit ethnic and socio-economic diversity within and between them, Arabic-speaking designation has been used as a surrogate marker in similar studies of physical and mental health to identify this target population and to assess this community [9, 10]. Further, language and cultural barriers are routinely identified as obstacles to accessing care and overcoming them have been demonstrated to improve health access for Arabic-speaking refugees in discrete vertical health programs such as breast-cancer screening [8, 10].

### Barriers facing refugees

Many refugees face challenges overcoming cultural and linguistic barriers within their new homes, especially as they attempt to access healthcare services [11]. Surveys of refugees in the United States have identified language and communication as common concerns among those attempting to access health services [7, 12–14]. Only 7% of refugees report “good” English proficiency during pre-arrival screenings [15], and similar concerns – along with a sense of social disconnection – are prevalent among Arab refugees and immigrants [16]. Furthermore, when compared to United States-born Arabs, Arab immigrants are more likely to self-report fair or poor levels of health – especially non-English speakers [17].

### Study purpose and objectives

The Refugee Medical Assistance program (RMA) provides short-term medical coverage for refugees and other persons who are eligible for Office of Refugees and Resettlement (ORR) benefits for eight months starting from the date of arrival in the country or granting of asylum [18]. There is little research on the health profile and healthcare satisfaction of this population after the end of the RMA period. This study seeks to use household interviews with Arabic-speaking refugees in Connecticut (CT) to identify and characterize the following after the end of the RMA period: barriers to their accessing health services; patterns of health-seeking behavior; prevalence of chronic disease as it relates to healthcare utilization; and acceptability of health services.

## Methods

### Study design

From January 1^st^ – December 31^st^, 2019, a cross-sectional observational study was conducted by surveying households of Arabic speaking refugees arriving in the state of Connecticut between January 2016 and June 2018.

### Participants

A contact list of 117 households representing 362 refugees and asylees was obtained from Integrated Refugee and Immigrant Services (IRIS) – the leading refugee agency in the State of Connecticut. Any household that identified as Arabic-speaking upon engagement with IRIS was included for contact in the study.

### Data collection

A team of 5 trained research assistants contacted each household for an interview up to five times before listing them as “unable to reach”. All known phone numbers for the household were contacted, as well as the number of a United States-based contact (frequently a family member, neighbor, or sponsor designated as a supplementary contact by the household). All research assistants were medical trainees and were proficient in Arabic language. Upon contact with a household member, verbal consent was obtained in Arabic or English at the discretion of the respondent, who was asked to respond on behalf of all members of the household. To help control for response bias and to ensure voluntarily participation, respondents were told that their responses would in no way impact their ability to continue to receive services at either IRIS or with their physicians. Then, an appointment was made to conduct the survey either in person or over the phone at the discretion of the respondent. If a phone survey was preferred, survey questions were also provided in writing in both Arabic and English to the respondent prior to the appointment. Households that completed the survey were given a VISA gift card worth $50 in consideration for their participation and to offset the cost of their time and transportation to the interview.

### Measures

The survey instrument was comprised of two parts. Part 1 included household demographics such as number of individuals in each household and their age, insurance status and type, patterns of recent health-seeking behavior, and information regarding chronic medical conditions and associated chronic medication use. For chronic medical conditions respondents were asked in lay-terms about household members with “high blood pressure”, “diabetes”, “heart problems”, “chronic difficulty in breathing or respiratory problems”, “high cholesterol”, “chronic headaches”, “blood related conditions”, “cancer”, and “other chronic conditions.” Part 2 assessed respondent satisfaction with health services using the Patient Satisfaction Questionnaire-18, a Likert scale questionnaire that evaluates seven domains of the healthcare including: general satisfaction, technical quality, interpersonal manner, communication, financial aspects, time spent with doctor, and accessibility and convenience (PSQ-18) with scores from 1.0 – 5.0 (RAND Corporation, Santa Monica, CA) [19] (Appendix – Survey Questionnaire). All responses were entered in a deidentified dataset using Qualtrics survey software (Qualtrics, Provo, UT).

### Analysis

Survey responses were analyzed using SAS/STAT® Software, version 9.4 (©2012 SAS Institute, INC., Cary, NC, USA) to generate descriptive statistics for the responses in both part 1 and part 2 of the survey. The significance level was set as *p* < 0.05, two-sided.

This study was approved by the Yale University Human Subjects Research Committee A HIPAA Waiver for written authorization was approved to allow authorization to be obtained verbally.

## Results

**Fig. 1:**
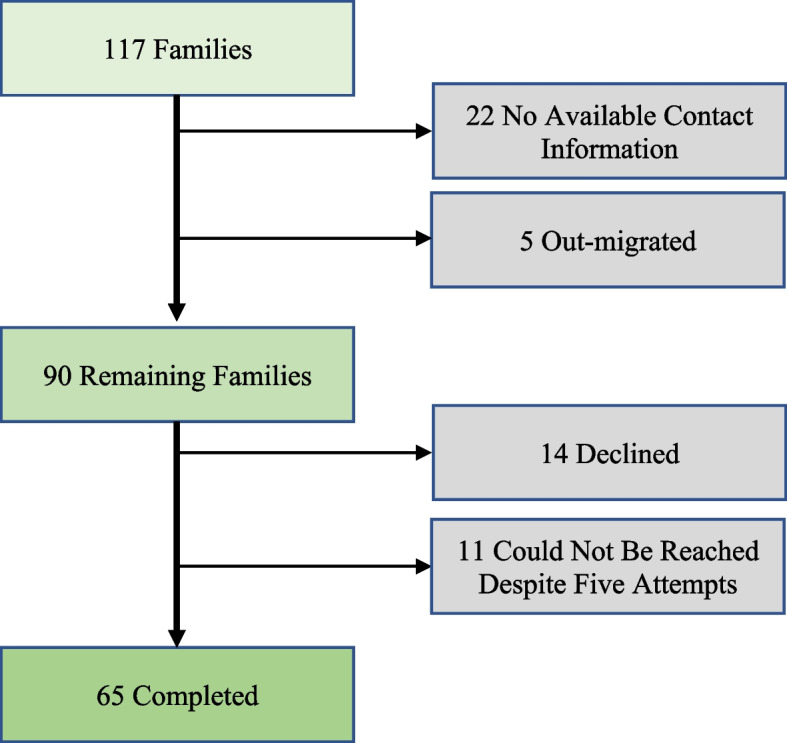
Survey participant composition

Of 117 refugee households identified that listed Arabic as their primary language, 5 were noted to have left the country, 22 had no available contact information, leaving 90 households who could be contacted for interview. Of these, 65 (72%) completed the survey corresponding to 295 individuals, 14 (16%) declined to participate, and 11 (12%) could not be contacted despite 5 separate attempts (Fig. 1 – Household Recruitment).

Sixty-one percent of Arabic-speaking refugee respondents were under age 25 years, primarily Syrian (72%) with a median household size of four (2 adults, 2 children) (Table 1). Despite the predominance of young persons and only 2% elderly, 47 households (72%) reported at least one family member with a chronic medical condition (Table 2). In addition, 25 household members (8%) reported functional limitation characterized as difficulty walking or needed assistance with activities of daily living (ADLs) (Table 2).

**Table 1:** Demographics, Arrival Year, Insurance Coverage, and Healthcare Utilization of Arabic-Speaking Refugees (*N* = 295) from 65 households arriving in Connecticut between 2016 and 2018

	**N**	**%**
**Females**	177	60%
**Age (years)**		
< 18	141	48%
18–24	38	13%
25–34	38	13%
35–55	41	14%
45–54	20	7%
55–64	11	4%
65 and older	6	2%
**Arrival Year by Household**		
2016	47	72%
2017	8	12%
2018	10	15%
**Country of Origin**		
Egypt	1	2%
Iraq	13	20%
Sudan	4	6%
Syria	47	72%
**Median Number of Adults per Household (Range)**	2	(1 – 6)
**Median Number of Children per Household (Range)**	2	(0 – 7)
**Covered by Health Insurance (Persons)**	285	97%
**Insurance Coverage(s) (No. Households)**		
Medicaid/Husky Alone	55	85%
Employer-Based Plan Alone	1	2%
Medicaid/Husky & Private Insurance	2	3%
Medicaid & Employer-based plan	5	8%
No Insurance	2	3%
**Medication Payment (No. Households)**		
Insurance	51	78%
Insurance & Out of Pocket	8	12%
Out of Pocket	5	8%
Insurance & Medications Purchased Abroad	1	2%
**Primary Care-seeking Venue (No. Households)**		
Primary doctor	53	82%
Walk in/ Urgent care	6	9%
Emergency department	5	8%
Other	1	2%
**No. Health Visits Prior 6 Months, Median**		
Primary Care	4	(0 – 100)
Walk-in Clinic/Urgent Care	0	(0 – 8)
Emergency Department	1	(0 – 10)
Other	0	(0 – 13)

**Table 2:** Difficulty with Activities of Daily Living, Medication Requirements and Accessibility, and Chronic Condition Prevalence as Compared to United States for Arabic-Speaking Refugees (65 Households representing 295 persons)

	**N**	**%**	
**Households by Number of Reported Chronic Medical Conditions**			
0	31	48%	
1	14	22%	
2	11	17%	
3	7	11%	
4	2	3%	
**Persons Reporting Chronic Conditions**			*USA Prevalence*^a^
Hypertension	19	6%	*29.9%*
Diabetes	17	6%	*9.8%*
Heart Disease	10	3%	*12.1%*
Chronic Respiratory Problems	21	7%	*6.2%, 9.5%*^b^
High Cholesterol	19	6%	*11.8%*
Chronic Headaches	16	5%	*15.3%*^c^
Blood-related condition (Hematologic)	7	2%	*5.6%*^d^
Cancer	1	0%	*9.4%*
Other Chronic Conditions	32	11%	–
**Difficulty with Activities of Daily Living (Persons)**	25	9%	
**Requiring Daily Medications (Persons)**	62	21%	
**Difficulty Obtaining Daily Medications**	17	6%	
**Medication Payment (No. Households)**			
Health Insurance	51	78%	
Health Insurance & Out of Pocket	8	12%	
Out of Pocket	5	8%	
Health Insurance & Medications Purchased Abroad	1	2%	

^a^Study data for % of total population of adults and children; Comparison Adult US prevalence drawn from [11–14]

^b^Reported metrics for COPD (6.7%), Asthma (9.5%)

^c^Migraine (15.3%)

^d^Anemia (5.6%)

Respondents reported 62 (21%) persons who required daily medication to manage chronic medical conditions and, of those, 17 (27%) reported difficulty acquiring needed medications. Most households, 51 (79%), reported that all household members used insurance to procure medications, while 9 households (14%) reported some household members using insurance while others paid for medications out-of-pocket, and 5 households (8%) reported that all household members paid for medication costs as out-of-pocket expense (Table 2).

Respondents reported a high level of health insurance coverage with 285 persons (97%) with some form of health insurance at least six months after the end of Refugee Medical Assistance (Table 1). Household members with health insurance were predominantly covered by Medicaid (United States government-sponsored health insurance program that is jointly funded by the Federal and State governments but administered by States to provide health insurance for low-income adults, children, pregnant women, and persons with disabilities [20]).Fifty-five households (85%) relied on Connecticut Husky State Medicaid as their sole form of health insurance, and an additional seven households (11%) relied on a mix of Medicaid and other forms of insurance such as private or employer-based plans. Only one household relied solely on an employer-based insurance and two households were uninsured.

Refugee households were integrated into the primary care system with 53 households (82%) that reported a primary care provider as their main method of obtaining health services. An additional 6 (9%) households primarily utilized episodic care in the form of urgent care and 5 households (8%) reported accessing the emergency department primarily for care. Households reported a median of 5 healthcare visits in the 6-months prior with 4 to the primary physician and one acute care visit (Table 2).

Refugee households reported a high median satisfaction care, albeit with very wide variation in each domain (Table 3). The PSQ-18 median scores by domain were General Satisfaction, 4.5 (1.5–5.0); Technical Quality, 4.0 (2.3–5.0); Interpersonal manner, 4.5 (2.5–5); Communication, 4.5 (2.0–5.0); Financial Aspects, 4.5 (1.0–5.0); Time spent with doctor, 4 (1.0–5.0); and Accessibility of services, 3.5 (1.8 – 5.0).

**Table 3:** PSQ-18 Scores by Domain for Arabic-Speaking Refugee Households in Connecticut between 2016^*^ and 2018(*N* = 65)

**Domain**	**Score**
**Psq: general satisfaction**	
Mean (SD)	4.02 (0.92)
Median (Range)	4.5 (1.5 – 5.0)
**Psq: technical quality**	
Mean (SD)	3.97 (0.65)
Median (Range)	4.0 (2.3 – 5.0)
**Psq: interpersonal manner**	
Mean (SD)	4.35 (0.72)
Median (Range)	4.5 (2.5 – 5.0)
**Psq: communication**	
Mean (SD)	4.09 (0.88)
Median (Range)	4.5 (2.0 – 5.0)
**Psq: financial aspects**	
Mean (SD)	4.07 (1.2)
Median (Range)	4.5 (1.0 – 5.0)
**Psq: time spent with doctor**	
Mean (SD)	4.02 (0.98)
Median (Range)	4.0 (1.0 – 5.0)
**Psq: accessibility and convenience**	
Mean (SD)	3.41 (0.77)
Median (Range)	3.5 (1.8 – 5.0)

*Includes one family that arrived last week 2015 but was aggregated with 2016 data

## Discussion

### Chronic medical conditions

Studies targeting adult refugee populations have documented similar rates of chronic non-communicable disease (NCD) as the United States population, with up to 51% of refugee adults having at least one NCD, and 9.5% having three or more NCDs [21, 22]. Specifically, rates of hypertension, diabetes, and hyperlipidemia in refugee populations, at 24.1%, 7.8%, and 27.1%, respectively, have been noted to be comparable to the United States population at large, however, these conditions were medically less controlled in the refugee population [23]. Despite similarities between refugee populations and the United States population at large, variation in the prevalence and types of chronic conditions among different refugee groups have been noted, with one study demonstrating significant differences in prevalence of chronic disease based on location of origin [24]. The amount of time living in the United States prior to interview could also impact rates of chronic conditions, with increases in both obesity and hypertension noted in refugees with increased length of stay in the United States [25].

Of 65 households surveyed, 47 households (72%) reported at least one member of the household had a chronic medical condition (Table 2). These results are limited by the lack of differentiation by respondents whether the chronic condition was reported for a child or an adult. Given that almost 50% of this population are children under 18, the reported rates for chronic conditions are a conservative estimate and the true rates among only adults may be twice the reported rates or higher. The Arabic-speaking refugee population in this survey was young, with an age breakdown similar to that of all refugees arriving in the same time period [26], but younger than that of the United States population at large. Forty-eight percent of refugees represented in this study were under the age of 18, compared to 24% for the United States population [27], and 61% were under the age of 25. Only 6 of the 295 (2%) Arabic-speaking refugees surveyed were over the age of 65. Further study into the age-adjusted prevalence of different chronic conditions among Arabic-speaking refugees could better contextualize the health challenges facing this population.

Further, 21% of respondents in our survey reported needing daily medications and 6% reported difficulty accessing medications. Our study was not designed nor powered to assess whether the difficulty accessing medications was due to refugee status, language barrier or Medicaid insurance. Numerous studies have cited difficulty in medication access among Medicaid-insured patients [28–30] although many of these are with respect to psychotropic medications and medications for treatment of substance use disorders. In those papers, dislocations resulting from changes in approved formularies and Medicaid regulations resulted in patients losing access to their prescribed medications. However, other studies including one from a natural experiment in Oregon [31] that analyzed poor patients’ access before and after Medicaid expansion revealed that Medicaid status resulted in higher access and adherence to prescribed medications and lower safety events from patients taking replacement medications or taking medications prescribed for another patient. The very high rate of health insurance in our study may indeed be a protective factor with respect to Arabic-speaking refugee access to medications while persistent difficulties with access may be related to health literacy and language barrier. Additional work that compares this population across states with differing enrollment eligibility may further elaborate these relationships.

### Health insurance coverage

Given this documented high prevalence of chronic NCDs amongst refugee populations in the United States, access to routine healthcare and daily medications to control chronic conditions is paramount to maintaining community health and reducing the cost of case management. In our study, Arabic-speaking refugee households were almost all covered by some form of health insurance – with most households covered by Medicaid. A similar result was found in the Iraqi refugee population in Michigan, where 100% of refugees surveyed one year after arrival were covered by Medicaid [32]. Conversely, 86.6% of refugees in San Antonio, Texas did not have any form of health insurance [33]. This variation in health coverage is likely attributable to differences in Medicaid eligibility requirements from state to state [34], with states adopting Medicaid expansion under the Patient Protection and Affordable Care Act, such as Michigan and Connecticut [35], providing increased access to healthcare for refugees in comparison to states that have not adopted the expansion, such as Texas. Connecticut has relatively broad inclusion criteria for Medicaid eligibility that includes all children under the age of 18, caretakers of children, and low-income adults under the age of 65 that meet income thresholds [36], contributing to a lower uninsured rate of 5% compared to the 9% uninsured rate for the United States [37]. The August 2019 Public Charge Final Rule aggressively interpreted the “likelihood of an immigrant becoming a public charge” resulting in widespread fear among refugees and other immigrant groups that applying for public services like food assistance or medical insurance could result in deportation or refusal of permanent residency or citizenship. One projection estimated that millions of children would lose health coverage as a result, with decreases in new applications and un-enrollments to maintain eligibility for citizenship [38]. United States Customs and Immigration Services stopped applying the rule on March 9^th^, 2021. The effect of this uncertainty in the intervening 18-month period that coincided with the onset of the COVID-19 pandemic on healthcare access for refugees and other immigrants groups warrants further study.

### Perceptions of healthcare and accessibility

Respondents reported high levels of median satisfaction as measured by the PSQ-18. The PSQ-18 is a validated instrument used to measure patient satisfaction and has been used in contexts ranging from primary care to specialty services in both in-patient and outpatient settings [39–42]. The very high levels of access to health insurance in our sample may have contributed to overall satisfaction as many barriers to access and payment for health services are mitigated by inclusion in the Medicaid program. While there were high *median* scores in each domain, there was wide variability in scores reported for each domain including several low outlier scores. Our study was not powered to conduct sub-group analyses to identify the factors associated with low score reports in each domain. Additional work is needed in the form of qualitative interviews or focus groups to better understand the reasons underlying these negative perceptions of care received. Studies with larger samples powered to perform sub-group analysis could also help understand how different factors associate with scores in each domain.

The domain with the lowest median score was accessibility of services. Similar findings were reported in another study that also used the PSQ-18 to survey Vietnamese refugees in the United States [43]. In that study, many respondents had favorable views of their healthcare but indicated that language barriers made it challenging to access care. Several factors may contribute to these barriers in accessing care, including acculturation, lack of reliable transportation, difficulty navigating the complex United States healthcare system, and language and communication barriers [12–14, 44, 45].

The difficulty acquiring prescription medications for refugees in this study despite widespread insurance coverage also warrants further study. A recent systematic review on access to prescription medication and pharmacy services among refugees in Australia found that while there was a paucity of research in this area, a wide variety of factors including language and cultural barriers, difficulty navigating the system for obtaining prescription medications (e.g. may be used to simply purchasing directly from a pharmacist), use of traditional medicine and medication non-adherence all contribute to decreased access to medications [46]. Finally, future study into pre-departure and post-arrival socioeconomic status and health literacy and understanding of the United States healthcare system could help contextualize whether these variables influence access to prescription medication or other United States health services more broadly.

### Limitations

This study has several important limitations that must be considered. While every attempt was made to contact refugee households, 27 of the original 117 households obtained from IRIS had no contact information or were known to have returned to their home countries. We had no baseline information on these households and cannot assess how similar they are to the respondent households. It may be that these households who are disconnected from the refugee resettlement agency are those that are having greater difficulty obtaining health insurance and accessing health services.

Further, while we report a lower prevalence of chronic condition in the *overall* respondent population the survey instrument did not clearly request the number of *adults* with chronic conditions and while the number of individuals with chronic medical conditions were reported, several may have had more than one condition. As such we cannot say with precision the exact number of adults with chronic medical conditions from these data. If the number of persons with chronic conditions are assumed to all be adults, then the numbers more closely mirror or exceed the rates in the host community (hypertension – 12.3% vs 27.6%; Diabetes – 11.0% vs 8.4%; chronic respiratory conditions – 13.6% vs COPD 6.2% or asthma 9.5%; hematologic disorders – 4.5% vs 2%). Reported rates of these conditions may have also been impacted by phrasing in the questionnaire. The survey did not use the Center for Medicaid Services list of chronic conditions [47], instead opting for more general descriptions such as “Heart problems” instead of ischemic heart disease. As such, some patients may have diagnosed with a condition but not know it was chronic given the question phrasing.

The high degree of health insurance coverage in our study may limit the ability to generalize to other settings with lower access to health insurance for refugees. Most refugee respondents in this study were covered by Medicaid, the eligibility requirements and quality of which vary from state to state [34]. Roughly 40% of refugees are in states that have not adopted Medicaid expansion under the Patient Protection and Affordable Care Act (USA) [48]. Studying PSQ-18 results among Arabic-speaking refugees with no insurance in these states may help reveal how geography of landing impacts perceptions and delivery of healthcare for refugees.

Additionally, the PSQ-18 was translated by the research team, all of whom are Arabic-native speakers with fluency in both colloquial and modern-standard Arabic but was not validated in Arabic prior to the study. There has since been a validated version of the instrument in Arabic that can be used [49].

Finally, our study collected responses in 2019 from families arriving between 2016 and 2018. We note that with the passing of United States Executive Order 13769 under the Trump administration, the total number of refugees admitted to the United States from the “Near East and South Asia” dropped almost 95% from a peak of 35,555 (2016) to a nadir of 1,999 (2020). This increased only slightly to 3,033 (2021) and 5,452 (2022) [50]. We believe that the overall profile of Arabic-speaking refugees in CT was unlikely to have changed drastically few recent arrivals but there may have been changes in health seeking behaviors related to the COVID-19 pandemic.

## Conclusion

Arabic-speaking refugees in CT in our study were young. A high percentage of refugee households reported coverage by health insurance and the majority reported state Medicaid as their primary insurance. Respondents expressed median high satisfaction with health services but with wide variation. Accessibility of health services in Arabic and ability to obtain medications remain areas in need of improvement for Arabic-speaking refugees in Connecticut. Additional research is needed to understand the factors that contribute to poor perceptions of health services in patients with this high access to health insurance. Furthermore, additional comparative research is needed to assess the impact of access to state-based health insurance on access to and perceptions of healthcare services for refugees.

## Supplementary Information

**Additional file 1: Appendix.** Survey instrument.

## Data Availability

The datasets of the current study are available from the corresponding author on reasonable request.

## Abbreviations

ADL: Activities of Daily Living
COPD: Chronic Obstructive Pulmonary Disease
CT: Connecticut
HIPAA: Health Insurance Portability and Accountability Act
IRIS: Integrated Refugee and Immigrant Services
MEDICAID: Medical insurance plan jointly funded by United States Federal and State governments to provide health services to low-income adults, children, pregnant women, and persons with disabilities
MENA: Middle East and North Africa
NCD: Non-communicable disease
ORR: Office of Refugee Resettlement
PSQ-18: Patient Satisfaction Questionnaire 18
RMA: Refugee Medical Assistance
UNHCR: United Nations High Commissioner for Refugees
USA: United States
